# Effect of LiNO_3_ on Expansion of Alkali–Silica Reaction in Rock Prisms and Concrete Microbars Prepared by Sandstone

**DOI:** 10.3390/ma12071150

**Published:** 2019-04-09

**Authors:** Jinxin Liu, Lanqing Yu, Min Deng

**Affiliations:** College of Materials Science and Engineering, Nanjing Tech University, Nanjing 210009, China; ljx.clnb@njtech.edu.cn (J.L.); yulanqing@njtech.edu.cn (L.Y.)

**Keywords:** microstructure, deformation, alkali–silica reaction, lithium nitrate

## Abstract

The aim of this research is to investigate the effect of LiNO_3_ on the alkali–silica reaction (ASR) expansion of reactive sandstone and the mechanism through which this occurs. This paper presents the results from tests carried out on rock prisms and concrete microbars prepared by sandstone and LiNO_3_. The findings show that LiNO_3_ does not decrease the expansion of these samples unless the molar ratio of [Li]/[Na + K] exceeds 1.66, and the expansion is greatly increased when its concentration is below this critical concentration. The expansion stress test proves that Li_2_SiO_3_ is obviously expansive. X-ray diffraction (XRD) and scanning electron microscope (SEM) results indicate that LiNO_3_ reacts with the microcrystalline quartz inside sandstone, inhibiting the formation of ASR gel, and the formation of Li_2_SiO_3_ causes larger expansion. A high concentration of LiNO_3_ might inhibit the ASR reaction in the early stages, and the formation of Li_2_SiO_3_ causes expansion and cracks in concrete after a long period of time.

## 1. Introduction

The alkali–silica reaction (ASR) is one of the main reasons for the decline of concrete durability. Following the discovery of the beneficial effects of lithium ions on ASR-related expansion in 1951, the effects of lithium compounds on ASR have been extensively studied [1]. However, the exact role of Li+ ions in controlling ASR is still unclear [2,3,4,5]. Several mechanisms have been proposed, including (i) enhance chemical stability of reactive silica exposed to pore solution [6,7,8], (ii) formation of less expansive Si–Li reaction products [9,10,11,12], (iii) formation of physical barrier by insoluble Si–Li reaction products [11,13,14]. However, the proposed mechanisms have mainly been determined experimentally under specific conditions and are not applicable to the most situations.

Lithium nitrate (LiNO_3_) is considered to be the most excellent compound for inhibiting ASR in various lithium-containing admixtures. Compared to other lithium-containing admixtures, the major advantage of LiNO_3_ is that it does not contribute to the increase of hydroxide (OH^−^) ion concentration, which eliminates the pessimum effects of other lithium-containing admixtures [15,16]. LiNO_3_ is also superior to other lithium-containing admixtures due to its neutrality, high solubility and good effect on concrete deformation. The use of LiNO_3_ as a lithium-containing admixture significantly reduces the expansion and affects the chemical composition and microstructure of the reaction product in mortar samples [11]. The addition of LiNO_3_ in the molar ratio of [Li]/[Na + K] at a level of 0.74 can generally reduce the expansion of mortar samples prepared with reactive aggregates to reach a safe and nondestructive level [17].

One of the unresolved issues related to the use of lithium-containing admixtures is related to the determination of the reaction product of the aggregate and the solution. Most previous studies did not directly confirm the existence of a production layer as a physical barrier, let alone explain the chemical composition, microstructure or effect of protection [18,19]. Feng X [20] considered that a mixed product made from the reaction of LiNO_3_ with aggregate included crystalline Li_2_SiO_3_ crystals and Li-containing low-Ca silica gel, thereby inhibiting the ASR reaction in concrete. Through MR experiments, Kim T [21] showed that the most likely mechanism to explain the excellent ability of Li+ ions to inhibit ASR involves the dense physical barrier, which is formed by the reaction products covers delimited areas on the exposed surface of the reactive aggregate. Zhou BF [22] found that quartz glass slices immersed in the alkaline solution with LiNO_3_ and Ca(OH)_2_ were well protected by a production layer consisting of Li_2_SiO_3_ crystals and CSH that densely and firmly covered the surfaces of samples, but seriously corroded in solutions with only LiNO_3_ or Ca(OH)_2_.

We studied the expansion of reactive aggregate with the rock prism test and concrete microbar test. XRD and SEM were used to find the reaction product to explain the effect of LiNO_3_ on the alkali–silica reaction of the reactive aggregate.

## 2. Materials and Methods

### 2.1. Aggregate

**Figure 1:**
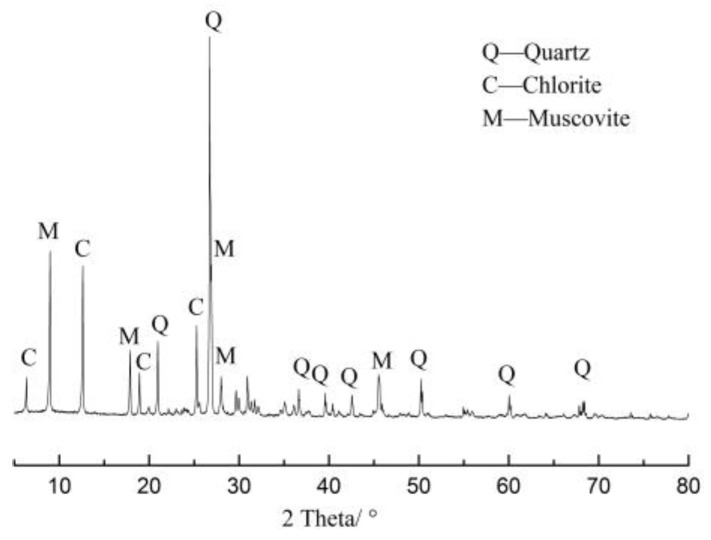
XRD pattern of sandstone.

Sandstone aggregate from the Lianghekou hydropower station in China was used. Figure 1 shows the XRD pattern of sandstone. The chemical composition of sandstone is shown in Table 1.

**Figure 2:**
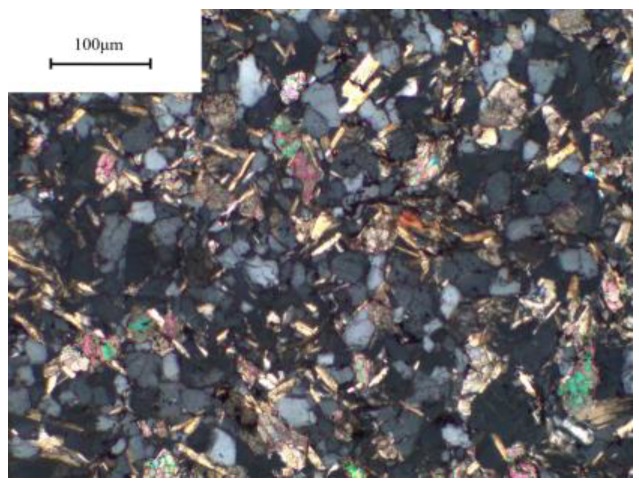
Microstructure of sandstone.

We can see a large amount of microcrystalline quartz in the sandstone from the polarizing microscope photograph Figure 2.

### 2.2. Rock Prism Test

The effect of LiNO_3_ on the expansion of sandstone in different alkali solutions was studied in accordance with ASTM C589. The sandstone was cut into the rock prisms 10 × 10 × 30 mm in size in the same direction. The nail heads were fixed on the ends of the rock prisms with P•II525 Portland cement, and the nail heads were covered at both ends with a damp cloth until the cement hardened. The hardened rock column was cured in clear water for 24 h. The length of every rock prism was measured to be the initial length of the rock prism by a spiral micrometer with an accuracy of 0.001 mm. Then the rock prisms were separately cured in different alkaline solutions at 80 °C. The curing solutions were mixed solutions of NaOH and LiNO_3_, and the ratios of Li/Na were 0, 0.41, 0.83, 1.24, 1.66, 2, and 3 respectively. The fresh curing solutions were replaced every 28 days. After curing to the set age, all the rock prisms were taken out and cooled to room temperature. Then, we measured the length and recorded the data to calculate the expansion rate at this time. 

### 2.3. Concrete Microbar Test

The concrete microbars of 40 mm × 40 mm × 160 mm in size had a cement: aggregate ratio of 1 and a water: cement ratio of 0.3 according to RILEM AAR-5. Sandstone aggregates with grain sizes of 5 or 10 mm were used. The alkali content of cement was adjusted to 2% Na_2_O equivalent by adding NaOH into the mixing water. LiNO_3_ was added to the mixing water in different molar ratios of lithium ions to alkali ions [Li]/[Na + K], such as 0, 0.41, 0.83, 1.24, 1.66, 2, and 3. The fresh curing solutions were replaced every 28 days. NaOH and LiNO_3_ were added to the curing solutions in the same molar ratio and the concentration of NaOH in every solution was 1 mol/L. After curing to the set age, all concrete microbars were taken out from the curing solutions and cooled to room temperature. Then we measured the length and recorded the data to calculate the expansion rate at this time.

### 2.4. Expansion Stress Test Apparatus 

**Figure 3:**
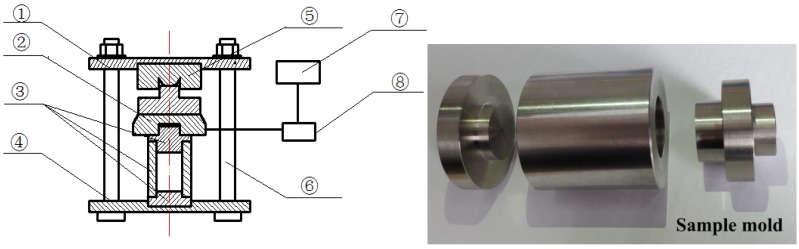
Schematic diagram of the expansion stress testing apparatus (①: top plate; ②: sensor; ③: sample mold; ④: bottom plate; ⑤: anti-load measuring head; ⑥: constrained screw; ⑦: data acquisition system; ⑧: transmitter).

The expansion stress caused by the activity of SiO_2_ and LiNO_3_ was tested by the expansion stress test apparatus which can be seen in Figure 3. We put 50 g SiO_2_ powder made of quartz glass with granules of less than 80 µm in size into the sample mold and compressed it into a compacted body by a press until the body could hold the pressure of 650 MPa for 5 s. Then, we put the mold into the expansion stress test apparatus with a pressure of 25 ± 0.1 MPa by tightening the nut. The apparatus was cured in a constant temperature curing box in a solution of 1 mol/L NaOH and 0.83 mol/L LiNO_3_ at 60 °C. Because the expansion of the rock prism and the concrete microbar samples greatly increased at an [Li]/[Na + K] dosages of 0.83 compared to the reference sample, the pressure sensor used in this device is suitable for temperature below 70 °C. The expansion stress was calculated according to Equation (1):

(1) $$\sigma =\frac{4(F_{t}-F_{0})g}{\pi d^{2}}$$

where *σ* is the expansion stress (MPa); *F_t_* is the sensor value at time *t* (kg); *F*_0_ is the initial value of the sensor (kg); *g* is the acceleration due to gravity and with a value of 9.8 m/s^2^; *d* is the inner diameter of the mold with a value of 24 mm; and the value of *π* is 3.14.

## 3. Results and Discussion

### 3.1. Rock Prism and Concrete Microbar Deformation

**Figure 4:**
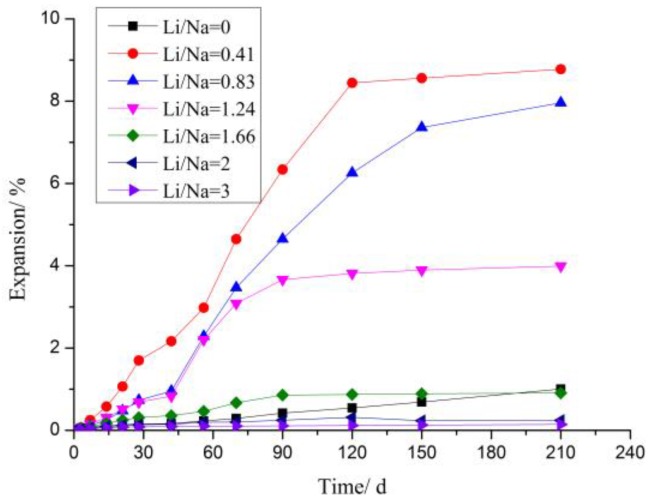
Expansion curves of rock prisms cured in different alkaline solutions at 80 °C.

Figure 4 showed the expansion curves of rock prisms cured in solutions with different concentrations of LiNO_3_ and 1 mol/L NaOH compared to the reference sample cured in 1 mol/L NaOH solution without lithium additive. LiNO_3_ added at Li/Na ratios of 0.41, 0.83, and 1.24 to solutions increased the expansion of samples obviously; the largest expansion was 8.8%, which caused damage to the samples. LiNO_3_ added at doses of 2 and 3 decreased the expansion of samples; samples expanded by about 0.25% in comparison to 0.91% in reference samples at 210 days. A dose of 1.66 decreased expansion after 150 days.

The results indicated that LiNO_3_ did not decrease the expansion of rock prisms until its concentration exceeded 1.66 mol/L and the expansion was greatly increased when its concentration was below this critical concentration. The expansion must be caused by the formation of other reaction products such as Li_2_SiO_3_ rather than just ASR. The data from the early days showed that the lower the Li concentration was, the faster the reaction was. The expansion curve of the sample at a dose of 0.41 tended to be gentle after 120 days indicating that the main reaction of the expansion had been completed or the expansion stress had been released from the cracks.

The rock prisms with large expansion at 210 days cracked or were even damaged, showing that the expansion of aggregate contributed greatly to the expansion of concrete made of sandstone. However, we studied the effect of LiNO_3_ on concrete microbars using the modified RILEM AAR-5 standard considering the directionality of rock expansion and the limitations of cement on aggregates.

**Figure 5:**
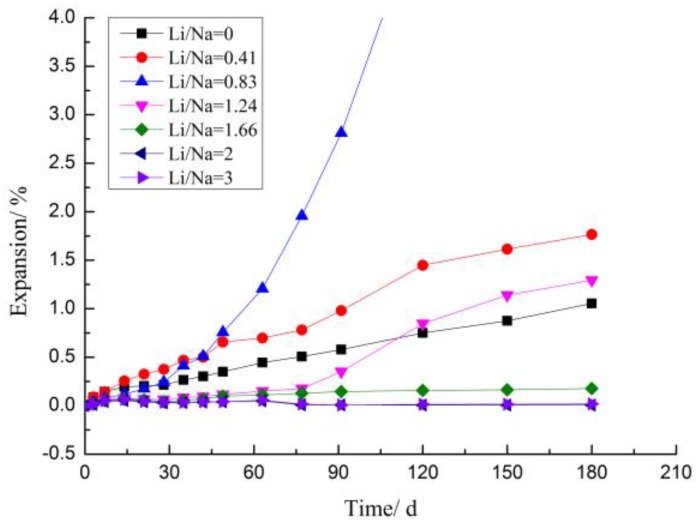
Expansion curves of concrete microbars cured in different alkaline solutions at 80 °C.

Figure 5 showed the expansion curves of mortar microbars prepared with LiNO_3_ compared to the reference sample prepared without lithium additive. The results indicated that critical Li concentration for concrete microbars was dose of 2. The samples with LiNO_3_ at [Li]/[Na + K] ratios of 0.41 and 0.83 increased expansion while samples at doses of 1.66, 2, and 3 decreased expansion in comparison to the reference samples. Doses of 1.66, 2, and 3 appeared to be effective in minimizing expansion over the time period tested and even made the samples micro-shrink so the expansion value of samples decreased from about 0.049% at 63 days to about 0.017% at 210 days, whereas at a dose of 0.83, expansion significantly increased after 60 days, and samples were damaged at 120 days. It was hard to make such a huge expansion with ASR gel, and we did not find ASR gel in the samples with LiNO_3_. So, the reason for expansion must be a different reaction from that of the general ASR.

At a dosage of 0.41, expansion showed an increasing trend with respect to the reference sample during the first 28d which indicated that the lower the concentration of Li^+^, the faster the reaction in the early stage. However, dose of 0.83 exhibited the larger expansion compared to dose of 0.41 after 47 days because large number of formed cracks increased the reaction area and dose of 0.83 provided more Li^+^.

The concentration of Li^+^ can be considered constant as the fresh solutions were replaced every 28 days. The dose of 1.24 appeared to be effective in reducing expansion during the first 80d, but the expansion increased rapidly after 80d and even was greater than the reference samples at 120d. This phenomenon may result from that the dose of 1.24 was so near the critical concentration that the reactions of increasing expansion occurred so slowly that cracks cannot be observed until samples were cured after several weeks. And with the constantly increasing of cracks, the increasing expansion reaction was more and more intense which led to the accelerated expansion in the later stage. So high concentration of Li^+^ cannot inhibit the expansion of mortar microbars after many years. And this phenomenon also proved that protective layer may not really exist, that is to say, the production layer may not be the real reason for decreasing the expansion of ASR.

**Figure 6:**
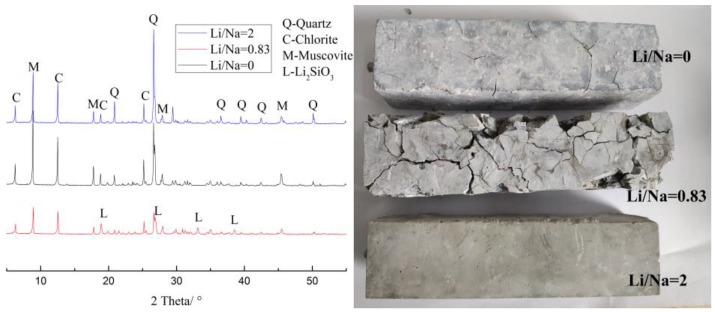
XRD patterns of aggregates in concrete microbars and images of corresponding concrete microbars cured in different alkaline solutions at 80 °C.

By studying the images displayed by the polarizing microscope, we found that the cracks were mainly formed inside the aggregate of the concrete microbars. To study the reaction products in concrete microbars cured in different alkaline solutions, we analyzed the internal aggregate after the reaction at 120 days using XRD. Figure 6 showed the effects of the LiNO_3_ concentration on the mineralogical composition of crystalline reaction products in concrete microbars cured in 1 mol/L NaOH solution. Li_2_SiO_3_ was the only crystalline compound when the LiNO_3_ concentration was 0.83 mol/L. But we did not find any products from XRD when the LiNO_3_ concentrations were 0 and 2 mol/L.

This phenomenon was consistent with the results of the macroscopic expansion experiments which showed that the expansion of samples with 0.83 mol/L LiNO_3_ was greater than that of samples with 0 and 2 mol/L LiNO_3_. So, the formation of Li_2_SiO_3_ appeared to be the reason for the expansion. When the LiNO_3_ concentration was higher than a certain concentration, the product of Li_2_SiO_3_ was greatly reduced but not prohibited, resulting in a sudden increase in expansion at a later stage. When the concentration of LiNO_3_ was particularly high, the later time might be very long and the products of Li_2_SiO_3_ were very small and difficult to find.

Therefore, we consider Li_2_SiO_3_ to be a deleterious product of the alkali–silica reaction. LiNO_3_ behaves similarly to other lithium compounds, such as hydroxide or carbonate and gives a pessimum effect.

### 3.2. Expansion Stress

**Figure 7:**
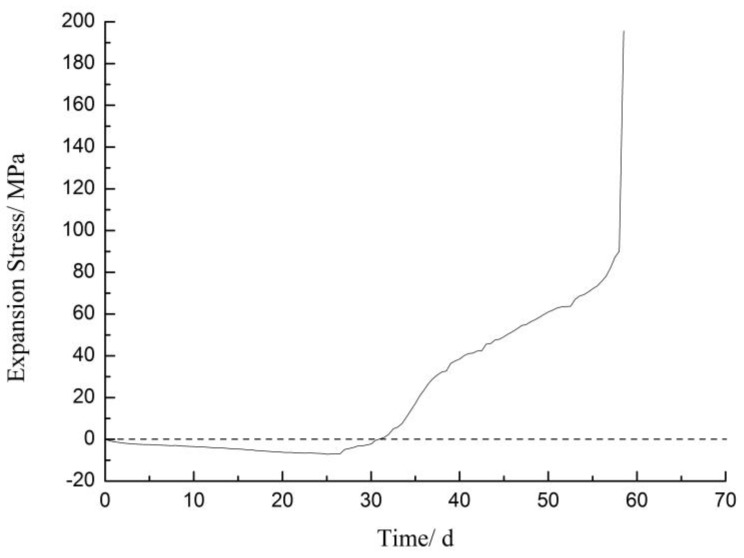
Expansion stress curve of the formed Li_2_SiO_3_.

Figure 7 showed that the expansion stress curve of a compacted body which made of SiO_2_ powder and placed in a self-made stress device. It proved that Li_2_SiO_3_ was obviously expansive. The curing solution included 0.83 mol/L LiNO_3_ and 1 mol/L NaOH, and the main reactions occurred as followed:

SiO_2_ + 2OH^−^ = H_2_O + SiO_3_^2−^ (2)

2Li^+^ + SiO_3_^2−^ = Li_2_SiO_3_. (3)

The expansion stress was continuously reduced until 27 days, and then it increased until 58 days due to the pre-stressing of the compacted body and the slow penetration of the solution. Finally, the stress broke out, resulting in damage to the device, and stress value was more than 195 MPa. The dissolution of SiO_2_ on the surface of compacted body caused formation of Li_2_SiO_3_, which was difficult for the surface of compacted body to absorb, resulted in a decrease in the expansion stress during the early days. Then, Li_2_SiO_3_ was formed inside, causing a rise in expansion stress as the solution continuously penetrated into the compacted body. The sudden rapid increase in stress on the last day may have been due to the filling of the internal voids of the compacted body and an order of magnitude change in the rate of reaction, which was similar to the rapid expansion of rock prisms and concrete microbars.

### 3.3. Microstructure

**Figure 8:**
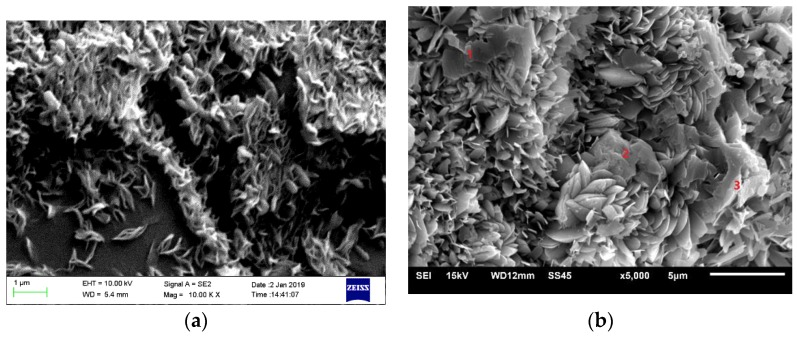
The SEM images of aggregate in concrete microbars cured in 1 mol/L NaOH with 0.83 mol/L LiNO_3_ at 80 °C: (**a**) at 42 days; (**b**) at 120 days.

Figure 8 presents the findings from the observations of the microstructure of mortar microbars cured in 1 mol/L NaOH with 0.83 mol/L LiNO_3_ at 80 °C. Figure 8a presented products formed at 42 days. Needle-like and filamentous crystals containing only Si and O represent Li_2_SiO_3_ formed on the surface of SiO_2_ around the cracks, because Li cannot be detected by energy dispersive spectrometer (EDS). Figure 8b presented products at 120 days. The many spindle-like crystals containing only Si and O represent Li_2_SiO_3_ which formed by the growth of needle-like and filamentous crystals. There were some SiO_2_ residues at position 1, 2, and 3, and cracks were mostly filled with products.

As mentioned before, LiNO_3_ did not decrease the expansion of samples until its concentration exceeded 1.66 mol/L. The expansion of samples when LiNO_3_ was present in concentrations below the critical concentration increased more quickly than in the reference sample. There must have been lots of formed products, namely, Li_2_SiO_3_ and ASR gel, leading to significant expansion of mortar microbars. However, it was hard to produce such a huge expansion with ASR gel, and we did not find ASR gel in the samples with LiNO_3_. It was indicated that Li^+^ reacted with the microcrystalline quartz inside the aggregate causing most of the expansion and this reaction consumed the reactants belonging to the ASR and inhibited the formation of ASR gel. Formed Li_2_SiO_3_ resulted in expansion increasing the reaction area, accelerated the reaction rate and finally formed larger cracks.

**Figure 9:**
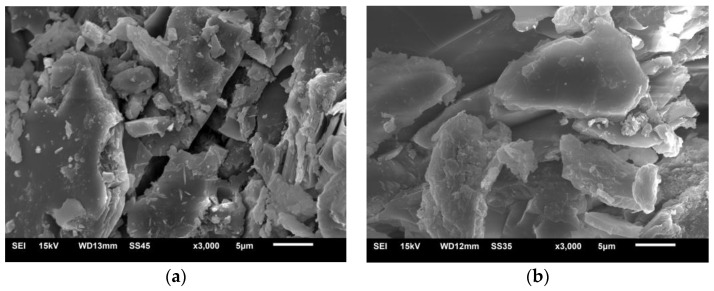
The SEM images of aggregate in concrete microbars cured in different alkali solutions at 80 °C: (**a**) 1 mol/L NaOH; (**b**) 1 mol/L NaOH and 2 mol/L LiNO_3_.

Figure 9 presented the findings from the observations of the microstructure of mortar microbars cured in 1 mol/L NaOH with or without 2 mol/L LiNO_3_ at 120 days. Compared with Figure 8, there were little reaction product and cracks in Figure 9a, which shows the sample in solution without LiNO_3_. The surface shown in Figure 9b with 2 mol/L LiNO_3_ was so smooth that there were almost no product formation and no cracks. Excess lithium nitrate made the sandstone denser, which corresponded to the minimal expansion and even micro-shrinkage of concrete microbars.

However, the mechanism by which LiNO_3_ control ASR when the concentration was beyond the critical concentration was still unclear, because the reaction product was too difficult to find. However, it is questionable as to whether that less expansive Si–ؘLi reaction products formed, because the product of Li_2_SiO_3_ was obviously expansive. Thus, it is worthwhile to continue our research to determine why Li_2_SiO_3_ did not form or formed less in concrete microbars with high concentrations of LiNO_3_. We believe the discovery of this reason will be of great value for determining the mechanism by which Li^+^ controls ASR.

## 4. Conclusions

1. LiNO_3_ did not decrease the expansion of rock prisms and concrete microbars with sandstone until the molar ratio of [Li]/[Na + K] exceeded 1.66, and expansion increased when the LiNO_3_ concentration was below the critical concentration.
2. The expansion stress test proved that Li_2_SiO_3_ is obviously expansive and the expansion stress was more than 195 MPa at the end of the test.
3. The XRD and SEM analyses indicated that product of Li_2_SiO_3_ caused greater expansion of samples, and reaction consumed the reactants belonging to ASR and inhibited the formation of ASR gel. LiNO_3_ reacted with the microcrystalline quartz inside the aggregate of sandstone and formed Li_2_SiO_3_; the expansion increased the reaction area, accelerated the reaction rate and finally, caused more and larger cracks.
4. The long-term effectiveness of excessive LiNO_3_ at inhibiting ASR was questionable. The high concentration of LiNO_3_ only inhibited the ASR reaction in the early stages and the formation of Li_2_SiO_3_ caused expansion and cracks in the concrete after a long period of time.

According to the results, the formation of Li_2_SiO_3_ caused greater expansion of sandstone when in the molar ratio of [Li]/[Na + K] was less than 1.66. LiSiO_3_ did not form or minimally formed in concrete microbars with higher concentrations of LiNO_3_, which should be studied further in the future.

## Figures and Tables

**Table 1:** Chemical composition of sandstone.

Oxide Component	SiO_2_	Al_2_O_3_	Fe_2_O_3_	CaO	MgO	K_2_O	Na_2_O	LOI.
Sandstone (wt%)	53.12	17.47	5.53	5.26	3.89	4.04	0.715	9.15

## References

[1] McCoy E.J., Caldwell A.G. (1951). New approach to inhibiting alkali-aggregate expansion. J. Am. Concr. Inst..

[2] Islam M.S., Ghafoori N. (2016). Experimental study and empirical modeling of lithium nitrate for alkali–silica reactivity. Constr. Build. Mater..

[3] Kurtis K.E., Monteiro P. (2003). Chemical additives to control expansion of ASR gel: Proposed mechanisms of control. J. Mater. Sci..

[4] Ohama Y., Demura K., Kakegawa M. Inhibiting ASR with chemical admixtures. Proceedings of the 8th International Conference Alkali–Aggregate Reaction.

[5] Tremblay C., Bérubé M.A., Fournier B., Thomas M.D.A., Folliard K.J. (2010). Experimental investigation of the mechanisms by which LiNO_3_ is effective against ASR. Cem. Concr. Res..

[6] Collins C.L., Ideker J.H., Willis G.S., Kurtis K.E. (2004). Examination of the effects of LiOH, LiCl, and LiNO_3_ on alkali–silica reaction. Cem. Concr. Res..

[7] Lawrence M., Vivian H.F. (1961). The reactions of various alkalis with silica. Aust. J. Appl. Sci..

[8] Wijnen P.W.J.G., Beelen T.P.M., Haan J.W., Rummens C.P.J., Ven L.J.M., Santen R.A. (1989). Silica gel dissolution in aqueous alkali metal hydroxides studied by 29Si NMR. J. Non Cryst. Solids..

[9] Diamond S., Ong S. The mechanisms of lithium effects on ASR. Proceedings of the 9th International Conference Alkali-Aggregate Reaction.

[10] Kawamura M., Lwahori K. (2004). ASR gel composition and expansive pressure in mortars under restrain. Cem. Concr. Compos..

[11] Leemann A., Lortscher L., Bernard L., Le Saout G., Lothenbach B., Espinosa-Marzal R.M. (2014). Mitigation of ASR by the use of LiNO_3_—Characterization of the reaction products. Cem. Concr. Res..

[12] Schneider J.F., Hasparyk N.P., Silva D.A., Monteiro P.J.M. (2008). Effect of lithium nitrate on the alkali–silica reaction gel. J. Am. Ceram. Soc..

[13] Feng X., Thomas M.D.A., Bremner T.W., Folliard K.J., Fournier B. (2010). New observations on the mechanism of lithium nitrate against alkali silica reaction (ASR). Cem. Concr. Res..

[14] Mitchell L.D., Beaudoin J.J., Grattan-Bellew P. (2004). The effects of lithium hydroxide solution on alkali silica reaction gels created with opal. Cem. Concr. Res..

[15] Diamond S. (1999). Unique response of LiNO_3_ as an alkali silica reaction-preventive admixture. Cem. Concr. Res..

[16] Stokes D.B., Wang H.H., Diamond S. A lithium-based admixture for ASR control that does not increase the pore solution pH. Proceedings of the 5th CANMET/ACI International Conference on Superplasticizers and Other Chemical Admixtures in Concrete, SP-173.

[17] Zapała-Sławeta J., Owsiak Z. (2016). The role of lithium compounds in mitigating alkali-gravel aggregate reaction. Constr. Build. Mater..

[18] Kawamura M., Fuwa H. (2003). Effects of lithium salts on ASR gel composition and expansion of mortars. Cem. Concr. Res..

[19] Berra M., Mangialardi T., Paolini A.E. (2003). Use of lithium compounds to prevent expansive alkali–silica reactivity in concrete. Adv. Cem. Based. Mater..

[20] Feng X., Thomas M.D.A., Bremner T.W., Folliard K.J., Fournier B. (2010). Summary of research on the effect of LiNO_3_ on alkali–silica reaction in new concrete. Cem. Concr. Res..

[21] Kim T., Olek J. (2016). The effects of lithium ions on chemical sequence of alkali–silica reaction. Cem. Concr. Res..

[22] Zhou B.F., Mao Z.Y., Deng M. (2018). Reaction of Quartz Glass in Lithium-Containing Alkaline Solutions with or without Ca.

